# Virus-producing cells determine the host protein profiles of HIV-1 virion cores

**DOI:** 10.1186/1742-4690-9-65

**Published:** 2012-08-13

**Authors:** Steven Santos, Yuri Obukhov, Sergei Nekhai, Michael Bukrinsky, Sergey Iordanskiy

**Affiliations:** 1 Department of Microbiology, Immunology and Tropical Medicine, George Washington University School of Medicine and Health Sciences, 2300 I Street NW, Ross Hall, Washington, DC, 20037, USA; 2 Department of Medicine, Center for Sickle Cell Disease, Howard University College of Medicine, 1840 7th Street, N.W, Washington, DC, 20001, USA; 3 Howard University College of Medicine, RCMI Proteomics Core Facility, 1840 7th Street, N.W, Washington, DC, 20001, USA

## Abstract

**Background:**

Upon HIV entry into target cells, viral cores are released and rearranged into reverse transcription complexes (RTCs), which support reverse transcription and also protect and transport viral cDNA to the site of integration. RTCs are composed of viral and cellular proteins that originate from both target and producer cells, the latter entering the target cell within the viral core. However, the proteome of HIV-1 viral cores in the context of the type of producer cells has not yet been characterized.

**Results:**

We examined the proteomic profiles of the cores purified from HIV-1 NL4-3 virions assembled in Sup-T1 cells (T lymphocytes), PMA and vitamin D_3_ activated THP1 (model of macrophages, mMΦ), and non-activated THP1 cells (model of monocytes, mMN) and assessed potential involvement of identified proteins in the early stages of infection using gene ontology information and data from genome-wide screens on proteins important for HIV-1 replication. We identified 202 cellular proteins incorporated in the viral cores (T cells: 125, mMΦ: 110, mMN: 90) with the overlap between these sets limited to 42 proteins. The groups of RNA binding (29), DNA binding (17), cytoskeleton (15), cytoskeleton regulation (21), chaperone (18), vesicular trafficking-associated (12) and ubiquitin-proteasome pathway-associated proteins (9) were most numerous. Cores of the virions from SupT1 cells contained twice as many RNA binding proteins as cores of THP1-derived virus, whereas cores of virions from mMΦ and mMN were enriched in components of cytoskeleton and vesicular transport machinery, most probably due to differences in virion assembly pathways between these cells. Spectra of chaperones, cytoskeletal proteins and ubiquitin-proteasome pathway components were similar between viral cores from different cell types, whereas DNA-binding and especially RNA-binding proteins were highly diverse. Western blot analysis showed that within the group of overlapping proteins, the level of incorporation of some RNA binding (RHA and HELIC2) and DNA binding proteins (MCM5 and Ku80) in the viral cores from T cells was higher than in the cores from both mMΦ and mMN and did not correlate with the abundance of these proteins in virus producing cells.

**Conclusions:**

Profiles of host proteins packaged in the cores of HIV-1 virions depend on the type of virus producing cell. The pool of proteins present in the cores of all virions is likely to contain factors important for viral functions. Incorporation ratio of certain RNA- and DNA-binding proteins suggests their more efficient, non-random packaging into virions in T cells than in mMΦ and mMN.

## Background

HIV-1 viral particles released from infected cells have been shown to incorporate many cellular proteins during the assembly and budding steps of morphogenesis. Findings from earlier studies, summarized in a web-based database (http://web.ncifcrf.gov/research/avp/protein_db.asp), identified more than three hundred cellular proteins in HIV-1 particles. HIV-1, as well as other lentiviruses, incorporates components of the cellular endosomal sorting machinery and cytoskeleton proteins involved in the process of particle assembly [1-5], surface proteins captured with the plasma membrane during budding [6,7], RNA-binding proteins associated with incorporated viral RNA and RNA-Gag complexes [4,8-10], chaperones [11], and multiple concomitant proteins (reviewed in [12]) whose functions in viral morphogenesis and infectivity are still unknown.

Upon fusion of an HIV-1 particle with a target cell, viral cores are released into the cytoplasm and rearranged into sub-viral particles called reverse transcription complexes (RTCs), which subsequently mature into pre-integration complexes (PICs). These nucleoprotein structures support reverse transcription and also protect and transport viral cDNA to the site of integration. RTCs are composed of both viral and cellular proteins. Since RTCs are formed from the viral cores, their initial composition is identical to that of viral cores. Other than the key enzymatic components, reverse transcriptase (RT) and integrase (IN), at least five other viral proteins involved in structural organization, cytoplasmic trafficking and nuclear import (matrix [MA], nucleocapsid [NC], capsid [CA], Nef and viral protein R [Vpr]), have been identified as components of HIV-1 RTCs [13-19], reviewed in [20-22].

Although the key early steps of HIV replication, reverse transcription and integration, are relatively autonomic, the participation of cellular proteins in early infection events has been demonstrated in previous studies [23-25]. After release from the viral particles, RTCs are still encapsulated in the shells formed by p24^CA^ molecules which are stable in the cytoplasm for at least several hours [26,27]. The shell is believed to protect the reverse transcription machinery and all encapsulated proteins from the cytoplasmic environment to provide optimal conditions for their functional activity [28] and may contribute to the nuclear import of PICs [19,29-31]. The shell likely limits the access of host cell proteins to the RTC interior. Thus, most cellular factors which may contribute to the functional competence of early RTCs should be expected to get into the complexes from the cores of infecting virions.

The cellular proteins, which are known to be hijacked by assembling virus particles from virus-producing cells and are involved in the early post-entry stages of HIV-1 infection, can be grouped into the following categories. (1) Factors involved in the spatial organization and correct folding of viral proteins in the virion and probably RTC: clathrin [5,32] and heat shock proteins (Hsp70, Hsc70, Hsp60) [11,33] are probably critical for spatial organization of Gag and Pol proteins and regulation of proteolytic processing and folding of the Pol products – RT and integrase; thioltransferase is found in HIV-1 virions and may be important for dimerization and activation of the viral protease [34], (reviewed in [12]); staufen1, an RNA-binding protein is packaged into virions and is involved in incorporation of HIV-1 RNA [8]. Interaction of staufen1 with Pr55^Gag^ zing finger motifs may also be important for Gag multimerization and formation of the viral capsids [35]. (2) Proteins which have an effect on cDNA synthesis/accumulation: lysyl-tRNA synthetase is incorporated through the interaction with Gag and is critical for the priming of reverse transcription [36]; uracil DNA glycosylase 2 (UNG2), a cellular DNA repair enzyme that binds HIV-1 integrase and Vpr [37,38] . The role of this enzyme in early post-entry steps of infection remains controversial. The hypothesis that the catalytic activity of Vpr-associated UNG could modulate virus mutation rate and APOBEC3G-mediated G-to-A hypermutations [39-41] was not supported by subsequent studies [42]. However, recently published work of Guenzel and co-authors showed that the virion-incorporated nuclear form of UNG2 facilitated reverse transcription through a non-enzymatic mechanism involving direct interaction with the p32 subunit of the replication protein A (RPA) complex [43]. RNA helicase A (RHA or DHX9) is packaged into HIV-1 virions probably through the interaction with an RNA or Gag polyprotein and facilitates reverse transcription [10]. The protein INI1/hSNF5, a member of SWI/SNF chromatin remodeling complex, has been shown to be packaged into virions through the direct binding to integrase [44], and is involved in the synthesis of reverse transcription products [45]. Later studies demonstrated that INI1/hSNF5 selectively recruits into HIV-1 virions the components of Sin3a-HDAC1 cellular complex, whose presence is critical for the early reverse transcription stage [46]. Furthermore, interaction of HIV-1 integrase with INI1 has been shown to be essential for the nucleosome remodeling of host chromatin and hence overcoming the structural nucleosome barrier for viral integration [47]. (3) Proteins involved in RTC formation, protection and transport: cyclophilin A, which is incorporated into virions via binding to the CA domain of Pr55Gag [23]. The role of CA-bound cyclophilin A in the viral life cycle is still unclear [48] Recently published data showed this protein to be critical for protection and stabilization of HIV-1 cores [49]. It may also be involved in PIC nuclear transport [31]. (4) Restriction factors of the early stages of HIV-1 infection: members of the APOBEC3 family of DNA/RNA editing cytidine deaminases, APOBEC3G (A3G) and APOBEC3F (A3F), are incorporated in Vif-negative virus particles (a small amount of these factors may be present also in Vif-positive virions) and then restrict reverse transcription by carrying out hypermutation of newly synthesized HIV-1 DNA [41,50-52]. Numerous studies have shown that A3G molecules from the target cell have no effect on cDNA deamination, and only virion-incorporated A3G affects viral DNA (reviewed in [53]), suggesting that the cDNA synthesis and accumulation machineries are effectively isolated from the environment of the target cell cytoplasm, but can be affected by factors which are encapsulated in cores and found within RTCs. Initiation of uncoating or disintegration of the capsid shell is believed to be dependent on the completion of reverse transcription [27,28]. Uncoated complexes containing viral cDNA are capable of interacting with numerous factors of the target cell, which are necessary for nuclear import and probably facilitation of viral genome integration (reviewed [21,54]).

Although numerous studies, including recently published proteomic analyses of whole highly-purified retroviral virions or virus-like particles, contain broad information about profiles of cellular proteins in viral particles [4,10,55,56], the host proteins associated specifically with the cores of mature HIV-1 virions have not been characterized. To identify the proteomic profiles of HIV-1 cores , we performed LC-MS/MS analysis of the cores isolated from virus particles of HIV-1 NL4-3 produced by Sup-T1 cells (T lymphocytes), PMA and vitamin D_3_ activated THP1 (model of monocyte-derived macrophages), and non-activated THP1 cells (model of monocytes), and Western blot analysis of selected proteins in the cores and producer cells. Potential involvement of identified proteins in the early stages of HIV-1 infection was assessed using gene ontology information and data from published genome-wide screens on proteins important for HIV-1 replication [57-59]. Our study revealed 202 proteins associated with HIV-1 cores. More than 20% of these proteins were detected in the cores of virions from all cell types, suggesting that this group contains cellular proteins potentially involved in viral replication. We found that some members of this group, which belong to subfamilies of RNA and DNA helicases, are packaged into the virions from producing T cells more efficiently than into virions from monocyte or MDM model cells, indicating that the mechanism of their incorporation is nonrandom.

## Results

### Preparation of HIV-1 core structures for proteomic analysis

Since HIV-1 is a highly variable virus, its different subtypes and individual variations may interact differently with cellular proteins [60]. Therefore, we selected the NL4-3 isolate of HIV-1 subtype B as a model virus for infection of different cell types: T lymphocytes and the model of monocytes and MDM. NL4-3 is a CXCR4-tropic isolate of HIV-1 that normally does not infect monocytes and macrophages, so to infect THP1 cells we used virus pseudotyped with amphotropic murine leukemia virus (MLV) Env glycoprotein. For isolation of viral cores and subsequent LC-MS/MS analysis, we used the virus particles produced by infected Sup-T1 cells (T lymphocyte cell line), THP1 (monocytic leukemia cells) and THP1 cells treated with phorbol 12-myristate 13-acetate (PMA) and vitamin D_3_. This treatment activates cell differentiation that results in acquisition of the biochemical and morphological characteristics of MDM. Thus, the activated THP1 cells may be considered as a model of macrophages [61,62].

**Figure 1:**
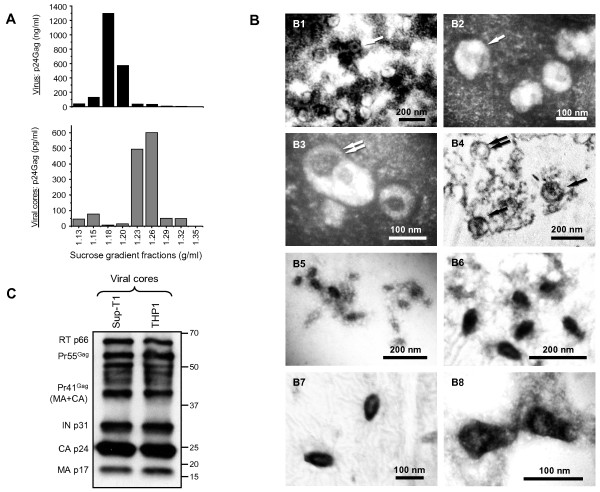
**“Spin-thru” purification isolates mature cores from HIV-1 virions.** The cores were isolated from the HIV-1 virions concentrated from culture media of infected Sup-T1, PMA-activated and non-activated THP1 cells by the “spin-thru” purification. **A** – CA p24^Gag^ profiles of 30-70% sucrose gradients after centrifugation of concentrated HIV-1 virions (upper panel) and “spin-thru”purified viral cores (lower panel). The 0.4 ml sucrose gradient fractions were collected, dialyzed against PBS and subjected to p24 ELISA. **B** – Electron microscopy of uranyl acetate negatively stained HIV-1 virions concentrated through 30% sucrose cushion (B1-B3), ultrathin sections of virions harvested from infected Sup-T1 cells (B4) and negatively stained core preparations after “spin-thru” purification (B5-B8). The negatively stained viral particles are indicated by single white arrows; extracellular vesicle contaminants in the preparations of concentrated virions are indicated by double white arrows; mature virions in the sections of viral preparatins are indicated by single black arrows, immature particles – by double black arrows. **C.** The cores of viruses produced by Sup-T1 and activated THP1 cells do not have differences in the profile of the GagPol processing products. The “spin-thru” purified and p24^Gag^ normalized cores were analyzed by Western blotting using the human HIV immunoglobulin (HIV-IG) from NIH AIDS Research & Reference Reagent Program. The bands of HIV-1 Gag and Pol processing products are indicated on the left.

To isolate core structures from HIV-1 virions produced by infected cells, we engaged a technique of “spin-thru” equilibrium density gradient sedimentation described earlier [63-66]. This method of ultra-speed centrifugation of previously concentrated HIV-1 virions through a sucrose density gradient overlaid with a detergent layer (1% Triton X-100) allows for the purification of mature lentiviral cores whose density varies from 1.23 to 1.27 g/ml [67,68] (Figure 1A, lower panel), whereas intact viral particles display buoyant density 1.18-1.20 mg/ml (Figure 1A, upper panel). To establish the purity of our viral core preparations from cellular vesicles, which have density similar to that of virions (1.14-1.20 g/ml) and may contaminate virus preparations [69], and to compare maturation of capsid cores in the viruses produced by T lymphocytes and MDM model cells, we engaged electron microscopy and Western blot analysis. Examination of the negatively stained concentrated virion samples in a transmission electron microscope revealed the presence of both extracellular vesicles and viral particles with diameter from 120 to 130 nm (Figure 1B1-B3). Analysis of ultrathin sections of the viral particles used for core isolation showed that the population represented a mix of immature (Figure 1B4, black double arrows) and mature virions (Figure 1B4, single arrow). The lipid membrane-covered structures were not found in purified viral core preparations. Only the conical-shape and oval structures with length about 80–110 nm were found in the samples of purified viral cores, indicating that the preparations after “spin-thru” purification contained only mature capsid cores (Figure 1B5-B8). Previous studies of cores from HIV-1 [64] and HIV-2 virions [63] isolated by “spin-thru” centrifugation method showed the presence of mature products of Gag and GagPol proteolytic processing (CA, MA, Vpx [for HIV-2] and RT with high enzymatic activity). Immunoblotting of our core preparations obtained after “spin-thru” purification of the same amounts of Sup-T1- and THP1-derived viral particles, carried out with human IgG prepared from pooled plasma of HIV antibody positive donors, revealed similar amounts of major products of Gag and GagPol processing, such as MA, CA, IN and RT proteins (Figure 1C). The Pr55^Gag^ and Pr41^Gag^ (MA + CA) have also been identified in the core samples from both viruses, suggesting that mature cores may contain some amounts of unprocessed Gag polyprotein. Taken together, our data indicate that the selected method of purification allows for the isolation of mostly mature cores from the pools of virions produced by both T lymphocytes and MDM model cells.

**Figure 2:**
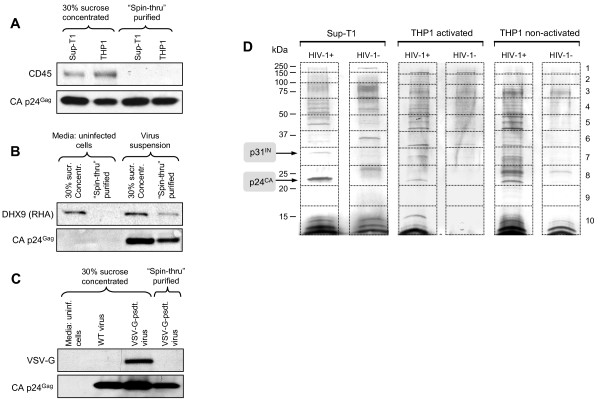
**“Spin-thru” purification allows isolation of viral cores for proteomic analysis. A, B** – “Spin-thru” purification separates viral cores from extracellular vesicles. Western blot analysis with anti-CD45 mouse monoclonal antibody of the virus samples before (left two lanes) and after (right two lanes) “spin-thru” purification. CD45 was used as an extracellular vesicle marker, p24^Gag^ as a reference viral protein (**A**). Western blot of culture media from uninfected 293 T/17 cells and suspension of virus harvested from pNL4-3 transfected 293 T/17 cells before and after “spin-thru” centrifugation with anti-RHA rabbit polyclonal antibody (**B**). **C** – “Spin-thru” purification separates viral cores from the envelope: Western blot analysis of VSV-G glycoprotein in the samples of VSV-G-pseudotyped HIV-1 before and after “spin-thru” centrifugation. **D** – Representative SDS-PAGE profiles of the preparations of purified virion cores. Coomassie blue-stained preparative SDS-PAGE 12.5% gels of HIV-1 viral cores and the cell culture supernatants of non-infected cells after “spin-thru” centrifugation were sectioned as shown by the dashed lines. Infected samples are marked as (+) and uninfected as (−). The molecular mass markers are indicated on the left and the gel fractions are specified on the right. Positions of HIV-1 core proteins p24^CA^ and p31^IN^ are shown on the left side.

We also performed Western blotting of our samples using the anti-CD45 antibody (Figure 2A). CD45 is known to be abundant in microvesicles, but is apparently excluded from HIV-1 virions [70]; the lack of this protein in our core preparations would confirm their purity from the vesicular fraction. Indeed, we did not detect CD45 in the samples of cores from HIV-1 virions produced by both Sup-T1 and THP1 cells (Figure 2A, two right lanes), whereas the specimens of culture media from these cells concentrated only through a 30% sucrose cushion contained detectable amounts of CD45. Western blotting of the samples of culture media from untransfected and NL4-3 proviral clone-transfected 293 T/17 cells using anti-RNA helicase A (RHA or DHX9) antibody showed presence of this DEAD box RNA helicase in the preparations of media from both transfected and untransfected cells after purification through 30% sucrose. However, in the “spin-thru” purified samples, RHA was detected only in the preparations of media containing HIV-1 (Figure 2B). Since RHA is known to be present in both vesicles [71] and HIV-1 virions [10,72,73], our analysis confirmed that the method of “spin-thru” centrifugation removed extracellular membranous structures from the 30% sucrose-concentrated cell culture supernatants, but retained intravirion core structures.

To further prove that “spin-thru” centrifugation purifies cores from intact virions, we tested the presence of VSV-G envelope protein in the samples of VSV-G-pseudotyped HIV-1 produced by co-transfected 293 T/17 cells after concentration through 30% sucrose cushion and “spin-thru” centrifugation (Figure 2C). The VSV-G was clearly detected in the samples of concentrated pseudotyped virus, but was not found in the core samples after “spin-thru” purification, confirming purity of the core preparations from the envelope glycoproteins.

The SDS-PAGE separation of our core preparations (Figure 2D) revealed major bands corresponding to proteins with molecular weights of 24 and 31 kDa (corresponding to HIV-1 CA and IN, respectively), indicating the presence of mature viral Gag and GagPol products in the analyzed core structures. On the other hand, multiple bands corresponding to the polypeptides of different molecular weights, which do not represent known HIV-1 proteins, suggest incorporation of many cellular proteins in the core structures of the viral particles produced by different cell types. The data of proteomic analysis (shown below) confirmed this suggestion. The staining of SDS-PAGE with Coomassie also revealed multiple protein bands in the control preparations, suggesting that the culture media from uninfected THP1 and especially Sup-T1 cells contained protein-rich, non-viral, non-membranous particles with buoyant density ≥1.23 mg/ml, probably the products of disintegrated dead cells (Figure 2D). Thus, to obtain proteomic profiles of the host proteins associated with HIV-1 viral cores, both viral cores and uninfected control preparations from each cell type were subjected to SDS-PAGE protein separation, trypsin digestion and subsequent LC-MS/MS analysis. The protein profile of each viral core sample was then compared with the corresponding control sample. Overlapping proteins were eliminated from the protein spectra of the viral cores, except the proteins whose scores were >5-fold higher in the preparations of viral cores than in control samples (proteins such as chaperones Hsp70 and Hsp90, and cytoskeletal proteins β actin, α and β tubulin, whose presence in core samples was confirmed by Western blot [Figure 4A]). As a result, a total of 202 cellular proteins were found to be associated with the cores of HIV-1 virions.

**Figure 3:**
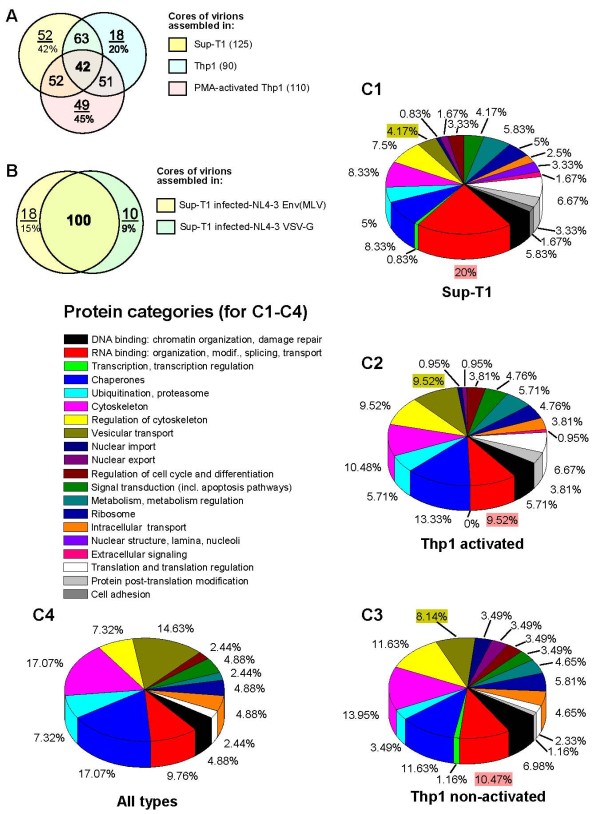
**Profiles of host proteins associated with the cores of HIV-1 virions from different producer cells – data of LC-MS/MS analysis. A, B** – Venn Diagrams depicting the number of overlapping cellular proteins within viral cores among the three producer cell types (**A**) and within the cores of viruses produced by the same cell type (Sup-T1) infected with HIV-1 NL4-3 strain pseudotyped with MLV Env (yellow) or VSV-G (green) envelope glycoproteins (**B**). The number of non-overlapping proteins and percent of these proteins within all cellular proteins identified in the core are shown as a numerator and denominator respectively. **C** – Categories of cellular proteins (by function) depicted as a percentage of the total proteins that were identified within the cores of virions produced by Sup-T1 (**C1**, n = 125), THP1 cells activated with PMA and vitamin D_3_ (**C2**, n = 110), and non-activated THP1 cells (**C3**, n = 90). The diagram of overlapping cellular proteins detected in all core preparations is shown on **C4** (n = 42).

### Proteomic profiling of HIV-1 viral cores

The proteins obtained from LC-MS/MS analysis of peptide preparations and filtered as described above and in *Materials and Methods* were categorized according to their functions and subcellular localization using NCBI protein database (http://www.ncbi.nlm.nih.gov/sites/entrez?db=Protein), NCBI RefSeq database (http://www.ncbi.nlm.nih.gov/RefSeq/) and DAVID Bioinformatics Resources 6.7 (NIAID NIH) (http://david.abcc.ncifcrf.gov). We compared our data with published results of the genome-wide analyses of cellular proteins involved in HIV-1 infection [57-59,74] and with global network of HIV-human protein–protein interactions [75] to assess the potential role of identified proteins in HIV-1 infection and putative mechanisms of their incorporation into the virion. The HIV-1 proteins identified in the viral cores are summarized in Table 1, the cellular proteins – in Tables2 and S1 in Additional file 1.

**Table 1:** **Viral proteins that were detected in viral cores of the virions produced by different types of infected cells**^a^

**Protein**	**Contained in samples from:**		
	**Sup-T1**	**Act. THP1**	**Non-act. THP1**
**Pr 160**^**GagPol**^ polyprotein-precursor (MA, CA, NC, p6, PR, RT, IN domains)	✓	✓	✓
**Pol TF** (p6*, PR, RT, IN domains)	✓	✓	✓
**Pol TF** (PR, RT domains)	✓	✓	
**RT p66 subunit**	✓	✓	✓
**Pr55**^**Gag**^ polyprotein-precursor ( MA, CA, sp2, NC, sp1, p6 domains) and probably partially-processed **Gag 48 kDa** polyprotein (MA, CA, sp2, NC domains)	✓	✓	✓
**RT p51 subunit**	✓	✓	✓
**gp41**^**TM**^**Env** glycoprotein	✓	✓	
**Gag 41 kDa** partially-processed polyprotein (MA, CA, sp2 domains)	✓		
**IN p31**^**Pol**^	✓	✓	✓
**CA p24**^**Gag**^	✓	✓	✓
**Nef**	✓	✓	✓
**Vif**	✓		
**MA p17**^**Gag**^	✓	✓	✓
**RNase H** 15 kDa Pol polypeptide	✓	✓	✓
**15 kDa partially-processed Gag precursor** (NC, sp1, p6)	✓		
**Rev**	✓		
**PR p10**^**Pol**^	✓	✓	✓
**NC p7**^**Gag**^	✓	✓	✓

^a^Proteins are indicated as being within the core from a specific producer cell type by a check symbol (✓).

Pol TF, polymerase trans-frame.

**Table 2:** **Cellular Proteins in HIV-1 cores**^a^

**Location**^**b**^	**Gene Name**^**c**^	**Accession Number**^**d**^	**Protein Name**^**e**^	**Contained in cores produced by:**		
				**SupT1**	**Act. THP1**	**N-Act. THP1**
***DNA Binding: Chromatin Organization, Replication, Topoisomerases***						
**N,C**	**RUVBL1**	**4506753**	**TATA binding protein interacting protein 49 kDa**	**✓**	**✓**	**✓**
**M,C,N**	**MCM5**	**1232079**	**Minichromosome maintenance complex component 5**	**✓**	**✓**	**✓**
N	RCC2	11360295	Regulator of chromosome condensation protein 2	✓	✓	
N,C	RUVBL2	5730023	TBP-interacting protein, 48-KD	✓		✓
N	TOP2B	288565	DNA topoisomerase II	✓		
N,C	SSRP1	4507241	Structure specific recognition protein 1	✓		
N	H2AFY	32492946	H2A histone family, member Y	✓		
N,C	ERVK-6	3600071	Reverse transcriptase encoded by human endogenous HERV-K retrovirus	✓		
N,C	MCM7	2134885	Replication licensing factor MCM7			✓
N,M	TEP1	1848277	telomerase-associated protein 1; TP-1			✓
N	HIST1H1B	4885381	H1 histone family, member 5; H1b			✓
N	HIST1H1C	4885375	H1 histone family, member 2; H1d			✓
*Number of Proteins per Cell Type:*				***6***	***3***	***4***
***DNA Damage Repair***						
**N**	**XRCC5**	**35038**	**Nuclear factor IV - KU80, ATP-dependant DNA helicase II**	**✓**	**✓**	**✓**
N	PRKDC	1362789	DNA-activated protein kinase		✓	✓
N	DDB1	12643730	DNA damage binding protein 1		✓	
*Number of Proteins per Cell Type:*				***1***	***3***	***2***
***DNA Binding: Transcription Regulation***						
N	POLR2B	23270691	Polymerase (RNA) II (DNA directed) polypeptide B			✓
N	RHOX11	27715523	Similar to Paired-like homeobox protein OTEX	✓		
*Number of Proteins per Cell Type:*				***1***	***0***	***1***
***RNA Binding: Structure Organization, Modification, Transport, Splicing***						
**N**	**UPF1**	**1575536**	**Regulator of nonsense transcript stability**	**✓**	**✓**	**✓**
**N**	**DHX9**	**3915658**	**ATP-dependent RNA helicase A - DEAD box protein 9**	**✓**	**✓**	**✓**
**N**	**EFTUD2**	**24474791**	**Small nuclear ribonucleoprotein component**	**✓**	**✓**	**✓**
**N**	**SNRNP200**	**14043179**	**Helicase hBrr2 200 kDa**	**✓**	**✓**	**✓**
N	HNRNPM	14141154	Heterogeneous nuclear ribonucleoprotein M isoform b	✓	✓	
N,C	EIF3A	32449796	Eukaryotic translation initiation factor 3, subunit A	✓	✓	
N,C	PABPC1	29743688	Poly(A) binding protein, cytoplasmic 1	✓	✓	
N	PRPF8	17999537	U5 snRNP-specific protein	✓		✓
N,C	HNRNPH1	5031753	Heterogeneous nuclear ribonucleoprotein H1	✓		✓
N	PDCD7	4416183	ES18 - U12-type spliceosome component	✓		✓
N,C	FLII	2135121	Flightless-I homolog	✓		✓
C	DDX3X	13514813	Helicase like protein 2 - DEAD/H box polypeptide 3	✓		
N,C,Mit	DDX17	5453840	RNA-dependent helicase p72 - DEAD box polypeptide 17 isoform 1	✓		
N	RENT1	1575536	Rregulator of nonsense transcript stability	✓		
N,C	SYNCRIP	26454828	Synaptotagmin-binding, cytoplasmic RNA-interacting protein	✓		
N,C	PABP2	12229876	Polyadenylate-binding protein 2	✓		
N,C	HNRNPR	13629286	Heterogeneous nuclear ribonucleoprotein R	✓		
N	SNRNP200	14043179	Small nuclear ribonucleoprotein 200 kDa (U5)	✓		
C	ACO1	9802308	Iron regulatory protein 1	✓		
C,M	FHL1	6942193	Four and a half LIM domains 1 protein isoform C	✓		
N	PCBP1	444021	Poly(rC) binding protein 1	✓		
N,C	HNRNPF	16876910	Heterogeneous nuclear ribonucleoprotein F	✓		
N,C	SRSF3	4506901	Splicing factor, arginine/serine-rich 3	✓		
N	RALY	27689091	Autoantigenic RNA binding protein		✓	
N	DDX21	11890755	RNA helicase II/Gu protein - DEAD box polypeptide 21		✓	
N,C,Mit	RTCD1	4506589	RNA 3'-terminal phosphate cyclase		✓	
N,C	HNRNPA1	30157273	Heterogeneous nuclear ribonucleoprotein A1		✓	
V,N,C	EIF5A2	9966867	Eukaryotic translation initiation factor 5A2; eIF-5A2 protein		✓	
N,C	SNUPN	6730226	D3b subcomplex of human core snRNP domain			✓
*Number of Proteins per Cell Type:*				***24***	***11***	***9***
***Cytoskeleton***						
**C**	**MYH10**	**1346640**	**Cellular myosin type B, heavy chain**	**✓**	**✓**	**✓**
**C**	**MYH9**	**29436380**	**Cellular myosin, heavy polypeptide 9**	**✓**	**✓**	**✓**
**C**	**TUBA1A**	**135395**	**Tubulin α 1**	**✓**	**✓**	**✓**
**C**	**TUBA1C**	**1438930**	**Tubulin α 6**	**✓**	**✓**	**✓**
**C**	**TUBB**	**1280489**	**Tubulin β 5**	**✓**	**✓**	**✓**
**C**	**TUBB3**	**1204535**	**Tubulin β 3**	**✓**	**✓**	**✓**
**C**	**ACTBL2**	**2973662**	**β Actin**	**✓**	**✓**	**✓**
C	TUBB4	2748197	β Tubulin 4Q	✓	✓	
C	TUBB1	13562114	Tubulin β 1	✓	✓	✓
C	TEKT1	16753231	Tektin 1	✓		
C	DYNC1H1	30581065	Dynein Heavy Chain, cytosolic		✓	✓
C	MYO1F	1924940	Myosin-IF		✓	✓
N,M	MYO1G	14269502	Unconventional myosin IG valine form		✓	
C,M	TUBG1	31543831	Tubulin γ 1			✓
C	TPM2	6573280	Tropomyosin 2 (β)			✓
*Number of Proteins per Cell Type:*				***10***	***11***	***12***
***Cytoskeleton Regulation***						
**C**	**HSPB1**	**662841**	**Heat shock protein 27**	**✓**	**✓**	**✓**
**M,N**	**MSN**	**14625824**	**Moesin**	**✓**	**✓**	**✓**
**C**	**CORO1A**	**5902134**	**Coronin, actin binding protein, 1A**	**✓**	**✓**	**✓**
M,C,V	CNP	180687	2',3'-cyclic-nucleotide 3'-phosphodiesterase; CNPase	✓	✓	
N,C	FLII	2135121	Flightless-I homolog	✓		✓
M,C	TTLL10	27663488	Tubulin tyrosine ligase-like family, member 10	✓		
C	ACTR2	27500905	Human ARP2 actin-related protein 2 homolog (yeast)	✓		
C,N	8-SEP	12654963	SEPT11 - Septin filament-forming cytoskeletal GTPase family member 11	✓		
N,C	PFN1	3891601	Human platelet profilin chain A	✓		
C,N	ACTR3	5031573	Human ARP3 actin-related protein 3 homolog (yeast)		✓	
C	DNAJA1	219588	DnaJ (Hsp40) homolog, subfamily A, member 1		✓	
C	RHOA	12654251	RAS homolog gene family, member A		✓	
	ARPC4-TTLL3	10436409	Actin related protein 2/3 complex, subunit 4-tubulin tyrosine ligase-like family, member 3 read-through fusion protein		✓	
C	ARPC4	15214920	Actin related protein 2/3 complex, subunit 4		✓	
C	RAC2	4506381	RAS-related C3 botulinum toxin substrate 2		✓	
M,V,C	TLN1	6739602	Talin			✓
M,C	FLNA	1203969	Filamin			✓
M	SPG8	20070788	Strumpellin			✓
C	NCKAP1L	32425702	HEM1 protein			✓
C	TBCD	13111855	Tubulin folding cofactor D			✓
M,C	CAP1	29739285	Human adenylyl cyclase-associated CAP protein homolog 1 (*S. cerevisiae, S. pombe*)			✓
*Number of Proteins per Cell Type:*				***9***	***10***	***10***
***Cell Signaling***						
**N,C**	**GNB2L1**	**5174447**	**Guanine nucleotide binding protein (G protein), beta polypeptide 2-like 1**	**✓**	**✓**	**✓**
	MYADM	27730943	Myeloid-associated differentiation marker		✓	✓
C,V	PTPRC	10999057	Protein tyrosine phosphatase		✓	✓
M	SEMA7A	3551779	Semaphorin L	✓		
M,C	PKN1	1085381	Serine/threonine protein kinase	✓		
C,M	MMP14	1705985	Matrix metalloproteinase 14		✓	
C	TIMP3	1304484	Tissue inhibitor of metalloproteinases-3		✓	
*Number of Proteins per Cell Type:*				***3***	***5***	***3***
***Nuclear Import***						
N,C	TNPO1	27681051	Karyopherin β2; importin β 2; transportin; transportin 1		✓	✓
V,N	TNPO3	6912734	Transportin-SR; importin 12; transportin-SR2	✓		
N,C	KPNB1	19923142	Karyopherin β1; importin 90; importin β-1			✓
N	KPNA2	1354365	Karyopherin α2; RAG cohort 1, importin α1			✓
*Number of Proteins per Cell Type:*				***1***	***1***	***3***
***Nuclear Export***						
N	XPO5	12407633	RANBP21/exportin 5	✓		✓
N,C	XPOT	17367977	Exportin T (tRNA exportin)	✓		✓
*Number of Proteins per Cell Type:*				***2***	***0***	***2***
***Apoptosis***						
**C**	**HP95**	**13375569**	**Programmed cell death 6 interacting protein**	**✓**	**✓**	**✓**
*Number of Proteins per Cell Type:*				***1***	***1***	***1***
***Extracellular Signaling***						
V,C,M	PTPRC	10999057	Protein tyrosine phosphatase, receptor type, C		✓	
C	AIMP1	27065983	Aminoacyl tRNA synthetase complex-interacting multifunctional protein 1	✓		
M	CLEC1A	30159086	C-type lectin domain family 1, member A	✓		
*Number of Proteins per Cell Type:*				***2***	***1***	***0***
***Intracellular Transport***						
**C,M**	**CLTC**	**30353925**	**Clathrin, heavy chain (Hc)**	**✓**	**✓**	**✓**
**M,C**	**RAB8A**	**234746**	**RAB8A, member RAS oncogene family**	**✓**	**✓**	**✓**
M	CPNE1	4503013	Copine I		✓	✓
C,N	TNIP1	1800305	TNFAIP3 interacting protein 1; HIV-1 Nef interacting protein	✓		
M,N,C	GC	18655422	Vitamin D binding protein		✓	
M,C	AP2B1	33504652	Adaptor-related protein complex 2, β1 subunit			✓
*Number of Proteins per Cell Type:*				***3***	***4***	***4***
***RNA Stability Regulation***						
C,N	RNH1	15029922	Ribonuclease/angiogenin inhibitor 1	✓		
*Number of Proteins per Cell Type:*				***1***	***0***	***0***
***Nuclear Lamina, intranuclear components***						
N	LMNB1	15126742	lamin B1	✓		
N	NCL	21750187	Nucleolin	✓		
N	DKC1	14602859	Dyskerin; dyskeratosis congenita 1	✓		
N	RPL3	18606060	Ribosomal protein L3	✓		
*Number of Proteins per Cell Type:*				***4***	***0***	***0***
***Cell adhesion***						
M,C	PCDHGA7	14196477	Protocadherin gamma subfamily A, 7	✓		
M	HABP2	4758502	Hyaluronan binding protein 2	✓		
*Number of Proteins per Cell Type:*				***2***	***0***	***0***
***Mitochondria***						
Mit	SLC25A6	113463	Solute carrier family 25 (mitochondrial carrier; adenine nucleotide translocator), member 6		✓	
*Number of Proteins per Cell Type:*				***0***	***1***	***0***
***Lipid Biosynthesis***						
C,N	FDFT1	11514495	Farnesyl-diphosphate farnesyltransferase 1	✓		
M,C	FASN	15779138	Fatty acid synthase		✓	
*Number of Proteins per Cell Type:*				***1***	***1***	***0***
***Transmembrane Ion Transport***						
N,M	SLC4A10	7513341	Sodium bicarbonate cotransport protein 2	✓		
C,M	ATP6V0E2	542837	ATPase, H + transporting V0 subunit e2		✓	
*Number of Proteins per Cell Type:*				***1***	***1***	***0***
***Cell-Cell Transport***						
M,C	ESYT1	7512911	Extended synaptotagmin-like protein 1		✓	
*Number of Proteins per Cell Type:*				***0***	***1***	***0***
***Ribosomal***						
**R**	**RPL7A**	**4506661**	**60 S-L7A**	**✓**	**✓**	**✓**
**R**	**RPL18A**	**11415026**	**60 S-L18A**	**✓**	**✓**	**✓**
R	RPL7A	17456110	60 S-L7	✓	✓	
R		27483402	40 S-S2	✓	✓	
R	RPL7L1	27498574	60 S-L7Like1	✓		✓
R	RPL13	15431295	60 S-L13	✓		✓
R	RPL24	4506619	60 S-L24		✓	✓
R	RPS8	4506743	40 S-S8	✓		
*Number of Proteins per Cell Type:*				***7***	***5***	***5***
***Ubiquitin/Proteasome***						
**C,N**	**UBA1**	**24485**	**Ubiquitin-like modifier activating enzyme 1**	**✓**	**✓**	**✓**
**C**	**BFAR**	**27675450**	**Bifunctional apoptosis regulator**	**✓**	**✓**	**✓**
**N,C**	**PSMC3**	**107855**	**Proteasome (prosome, macropain) 26 S subunit, ATPase, 3**	**✓**	**✓**	**✓**
C	PSMD7	2134660	Proteasome (prosome, macropain) 26 S subunit, non-ATPase, 7	✓		
M,C	PSMB1	12653473	Proteasome (prosome, macropain) subunit, β type, 1	✓		
N,C	PSMD11	2150046	Proteasome (prosome, macropain) 26 S subunit, non-ATPase, 11	✓		
C,N	PAAF1	33150632	Proteasomal ATPase-associated factor 1		✓	
C,N	RPN1	14124942	Ribophorin I		✓	
N,C	PSMD3	16550621	Proteasome (prosome, macropain) 26 S subunit, non-ATPase, 3		✓	
*Number of Proteins per Cell Type:*				***6***	***6***	***3***
***Metabolism, Metabolism Regulation***						
**M**	**PFKP**	**11321601**	**Phosphofructokinase, platelet**	**✓**	**✓**	**✓**
C,N,M	ACLY	13623199	ATP citrate lyase		✓	✓
M,C	PYGL	10120741	Chain A, Human Liver Glycogen Phosphorylase A		✓	✓
N,C	GPT	1507680	Glutamic-pyruvate transaminase (alanine aminotransferase)	✓		
C,M	PKM2	125604	Pyruvate kinase, M2 isozyme	✓		
C	SLC2A1	3387905	Solute carrier family 2 (facilitated glucose transporter), member 1	✓		
N,C	TPI1	16877874	Triosephosphate isomerase 1		✓	
*Number of Proteins per Cell Type:*				***4***	***4***	***3***
***Cell Cycle Regulation/Cell Differentiation***						
**N**	**CCNB3**	**14719420**	**Cyclin B3 isoform 3**	**✓**	**✓**	**✓**
C	PPP2R1A	21749746	Protein phosphatase 2A, regulatory subunit A, α	✓		✓
C,M	RAP1B	12751117	RAP1B, member of RAS oncogene family		✓	✓
C,N	PPP2R3B	7019501	Protein phosphatase 2A, regulatory subunit B'', β; PP2A B''	✓		
N,C	CDK11A	16357490	Cyclin-dependent kinase 11A	✓		
C	PRDX3	32483377	Peroxiredoxin 3 isoform b		✓	
C	TIMP3	1304484	TIMP metallopeptidase inhibitor 3		✓	
*Number of Proteins per Cell Type:*				***4***	***4***	***3***
***Nucleotide Biosynthesis***						
C	CAD	18105007	Carbamoyl-phosphate synthetase 2, aspartate transcarbamylase, and dihydroorotase	✓		
*Number of Proteins per Cell Type:*				***1***	***0***	***0***
***Amino Acid Biosynthesis***						
M,C,N	PHGDH	5771521	3-phosphoglycerate dehydrogenase	✓		
C	ASS1	16950633	Argininosuccinate synthase 1		✓	
C	GCN1L1	2282576	HsGCN1; GCN1 general control of amino-acid synthesis 1-like 1 (human homolog of yeast)			✓
*Number of Proteins per Cell Type:*				***1***	***1***	***1***
***Vesicular Transport***						
**C,M**	**CLTC**	**30353925**	**Clathrin**	**✓**	**✓**	**✓**
**C,M,V**	**COPA**	**4758030**	**Coatomer protein complex, subunit α; alpha coat protein; xenin**	**✓**	**✓**	**✓**
**V,C**	**MVP**	**19913410**	**Major Vault Protein**	**✓**	**✓**	**✓**
**C,V**	**TFRC**	**4507457**	**Transferrin receptor (p90, CD71)**	**✓**	**✓**	**✓**
**C,V**	**RAB7A**	**1709999**	**RAB7A, member RAS oncogene family**	**✓**	**✓**	**✓**
**C**	**PDCD6IP**	**13375569**	**Programmed cell death 6 interacting protein, HP95, AIP1/ALIX**	**✓**	**✓**	**✓**
C	RAB5C	4759020	RAB5C, member RAS oncogene family		✓	✓
C,V	EHD4	7212811	EH-domain containing 4		✓	
C	SAR1A	21634445	GTP-binding protein Sara; SAR1 homolog A (*S. cerevisiae*)		✓	
M,V	ARFRP1	1065361	Chain A, Human Adp-ribosylation factor related protein 1		✓	
V,N	ATP6V0A1	1638835	ATPase, H + transporting, lysosomal V0 subunit a1		✓	
C	RAB11A	4758984	RAB11A, member RAS oncogene family			✓
*Number of Proteins per Cell Type:*				***5***	***10***	***7***
***Aminoacyl tRNA synthetases***						
**C,N**	**IARS**	**31873336**	**Isoleucyl-tRNA synthetase**	**✓**	**✓**	**✓**
C,N	MARS	15929104	Methionyl-tRNA synthetase	✓		✓
C,N	RARS	2118344	Arginyl-tRNA synthetase	✓		
C,N	GARS	3845409	Glycyl-tRNA synthetase	✓		
C,Mit	WARS	8439415	Tryptophanyl-tRNA synthetase	✓		
C,N	DARS	4557513	Aspartyl-tRNA synthetase		✓	
C,N	QARS	11493441	Glutaminyl-tRNA synthetase		✓	
*Number of Proteins per Cell Type:*				***5***	***3***	***2***
***Translation and Translation Regulation***						
N,C	EIF3A	32449796	Eukaryotic translation initiation factor 3, subunit A	✓	✓	
C	EEF2	4503483	Eukaryotic translation elongation factor 2; polypeptidyl-tRNA translocase	✓		
C	CC2D1B	27715655	Coiled-coil and C2 domain containing 1B	✓		
C	CYFIP1	24307969	Cytoplasmic FMR1 interacting protein 1 (Sra1)		✓	
C	AIMP2	27662300	Aminoacyl tRNA synthetase complex-interacting multifunctional protein 2		✓	
V,N,C	EIF5A2	9966867	Eukaryotic translation initiation factor 5A2; eIF-5A2 protein		✓	
*Number of Proteins per Cell Type:*				***3***	***4***	***0***
***Protein Post-Translation Modification***						
C,M	PAFAH1B	4505587	Platelet-activating factor acetylhydrolase 1b, catalytic subunit 3	✓		
N,C	PARP1	130781	Poly [ADP-ribose] polymerase-1	✓		
C	USP14	4827050	Ubiquitin specific protease 14	✓		
C,N	SERPINC1	4502261	Serpin peptidase inhibitor, clade C (antithrombin), member 1	✓		
M,C	LPL	15030193	Lipoprotein lipase		✓	
M,C	NMT1	345862	N-myristoyltransferase 1		✓	
C,M	CPD	21903712	Carboxypeptidase D		✓	
*Number of Proteins per Cell Type:*				***4***	***3***	***0***
***Protein Degradation (non-proteasomal)***						
M	ANPEP	28678	Alanyl (membrane) aminopeptidase		✓	
*Number of Proteins per Cell Type:*				***0***	***1***	***1***
***Chaperones/Molecular Folding***						
**C**	**DNAJC13**	**7513063**	**DnaJ (Hsp40) homolog, subfamily C, member 13**	✓	✓	✓
**C**	**HSP90AB1**	**20149594**	**Heat shock protein 90 kDa α (cytosolic), class B member 1**	✓	✓	✓
**C,N**	**HSPA8**	**24234686**	**Heat shock 70 kDa protein 8**	✓	✓	✓
**C,M, N**	**CCT3**	**2136253**	**Chaperonin containing t-complex polypeptide 1 (TCP1), subunit 3 (γ); TCP1 ring complex protein TRiC5**	✓	✓	✓
**C,M**	**CCT6A**	**4502643**	**Chaperonin containing t-complex polypeptide 1 (TCP1), subunit 6A (ζ1)**	✓	✓	✓
**C,M**	**CCT6B**	**22654293**	**Chaperonin containing t-complex polypeptide 1 (TCP1), subunit ζ2**	✓	✓	✓
**C**	**HSPB1**	**662841**	**Heat shock 27 kDa protein 1**	✓	✓	✓
**C**	**PPIA**	**2624881**	**Human Cyclophilin A**	✓	✓	✓
N	NAP1L4	5174613	Nucleosome assembly protein 1-like 4; nucleosome assembly protein 2	✓		✓
N,M,C	TCP1	13540473	t-complex polypeptide 1 (TCP1)	✓		✓
N,C	CCT5	12804225	Chaperonin containing t-complex polypeptide 1 (TCP1), subunit 5 (ϵ)	✓		
C	P4HB	20070125	Prolyl 4-hydroxylase, β polypeptide		✓	
C	CCT7	5453607	Chaperonin containing t-complex polypeptide 1 (TCP1), subunit η		✓	
C	SERPINH1	123576	Serpin peptidase inhibitor, clade H (heat shock protein 47), member 1		✓	
C	HYOU1	5453832	Oxygen regulated protein 1		✓	
C	CANX	10716563	Calnexin		✓	
Mit	HSPA9	12653415	Heat shock 70 kDa protein 9 (mortalin)		✓	
C,N	CCT4	2559008	Chaperonin containing t-complex polypeptide 1 (TCP1), subunit δ			✓
*Number of Proteins per Cell Type:*				***10***	***13***	***10***

^a^ Cellular proteins were detected in the cores from the virions produced by different types of infected cells. Proteins are listed by functional category and indicated as being within the core from a specific producer type by a check symbol (✓). Bolded proteins were found in viral cores produced by all producer cells.

^b^ The locations of listed proteins are abbreviated as follows: C – Cytoplasmic, N – Nucleus, M – Plasma Membrane, Mit – Mitochondria, R – Endoplasmic Reticulum, V – Vesicles.

^c^ Official Gene Symbol as listed by HGNC.

^d^ Swiss-Prot Protein Accession number.

^e^ Full Protein name as listed in NCBI Protein Database (http://www.ncbi.nlm.nih.gov/protein).

### Viral proteins

The major proteins constituting the HIV-1 nucleocapsid core, CA, NC, IN and RT (both p51 and p66 subunits) were identified in the preparations from virions generated by all cell types (Table 1). All core samples also contained MA protein, which is located in retroviral virions mostly outside the capsid and forms a matrix between the viral capsid and envelope [76,77]. However, presence of MA in the RTCs and PICs [13,14,17,78,79] suggests that this protein is physically associated with the cores of HIV-1 virions. Comparison of our list of identified viral proteins with the MS/MS analysis data of whole MDM-produced HIV-1 particles [4] shows characteristic differences between the protein profiles of viral cores and whole virions. The gp120^Env^ glycoprotein detected in whole virions is absent in our samples; however, the gp41 transmembrane (TM) Env product was identified in the cores from virions assembled in T lymphocytes and MDM-like activated THP1 cells. Since gp41 has been found to be associated with MA during virion assembly and probably in mature viral particles [80,81] (reviewed in [82]), a low amount of this glycoprotein in detergent-purified core preparations was expected. The fact that we could not identify gp41 in the cores of virions from non-activated THP1 cells by LC-MS/MS confirms our suggestion that the concentration of this glycoprotein in our preparations is negligible. The absence of gp120^Env^ in our samples confirms purity of isolated core structures from the viral envelope and intact virions, shown also in Figure 2C.

The Tat, Nef and Vif proteins were identified earlier in the viral particles produced by infected MDM using proteomic methods [4]. We did not detect Tat in our core preparations, but MS/MS analysis revealed Nef in the viral cores from all analyzed cell types. Earlier studies indicated that Nef is incorporated into viral particles, stably associates with virion cores and facilitates reverse transcription and early steps of replication [16,64,83,84]. The Vif and Rev proteins were identified with low scores only in the cores from T lymphocyte-derived virus, suggesting their very low concentrations in the cores. HIV-1 Vif has been shown to interact with Pr55^Gag^ and viral protease during the assembly of virus particles [85]. Rev protein can be incorporated into cores in the complex with viral RNA. Surprisingly, we did not identify Vpr in the viral core samples. Numerous studies revealed Vpr in HIV-1 virions [86-88] and RTCs [15,89-91]. However, while traditional methods of identification using specific antibodies recognize Vpr in HIV-1 particles, LC-MS/MS analysis performed by Chertova and co-authors [4] also did not reveal this protein in the preparations of whole highly-purified virions. The visibility of protein for LC-MS is defined by a few factors, including affinity to C18 column (hydrophobicity) and ionization efficiency of the peptides. Typically, about 10% of tryptic peptides of a long protein are visible. Thus, some proteins could be invisible for LC-MS/MS in spite of overall high sensitivity of a method. Moreover, although trypsin cleaves Vpr to 14 peptides, most of them are not charged or have a negative total charge in solution with neutral pH (analyzed using Innovagen Peptide property calculator tool http://www.innovagen.se/), which may cause additional problem with their detection by mass spectrometry.

HIV-1 protease (PR) was observed in all our core preparations. At the same time, detection of Gag and GagPol polyprotein-precursors in respective gel fractions suggests that not all precursor molecules are subjected to the proteolytic cleavage during the virion assembly and a subset of unprocessed polyproteins is present in the mature viral cores.

### Cellular proteins

Within 202 unique cellular proteins revealed in our preparations of purified cores from HIV-1 virions, the samples from Sup-T1-derived virus included 125 proteins, while the virion cores from activated and non-activated THP1 cells contained 110 and 90 proteins, respectively (Table 2). A similar number of common proteins was found between cores from viruses produced by activated and non-activated THP1 cells (51), non-activated THP1 and Sup-T1 cells (63), and activated THP1 and Sup-T1 (52) (Figure 3A). Forty two proteins were common to all viral cores, which equates to 34%, 38% and 47% of the proteins found in the cores of viruses derived from Sup-T1, activated and non-activated THP1 cells, respectively.

We identified 125 host cell proteins in the cores of virions assembled in Sup-T1 cells; cores of the viruses from activated THP1 cells contained 12% less host proteins, whereas the number of host proteins in the cores of non-activated THP1-derived virus was 28% less than in the cores of virus from Sup-T1. Interestingly, only 20% of all cellular proteins identified in the cores of virus from non-activated monocytic cells were unique, other proteins were also found in the cores of the virions derived from Sup-T1 or activated THP1 cells. The cores of activated THP1 and Sup-T1-derived viruses, in contrast, contained 45% and 42% of unique host proteins, respectively (Figure 3A). Since the T lymphocytes and MDM (activated THP1 is a model of MDM) naturally support productive HIV-1 infection, whereas the non-differentiated monocytes may serve as HIV-1 reservoirs, but do not support active replication (reviewed in [92]), the incorporation of a smaller number of cellular proteins by the virions released from non-activated THP1 is probably related to the lower efficiency of virion assembly in this type of cells.

To validate the credibility of differences in protein composition between viral cores from different cell lines, we performed proteomic analysis of two core preparations purified from the same cell line. Cores of virions harvested from Sup-T1 cells infected with MLV Env-pseudotyped and VSV-G-pseudotyped NL4-3 viruses were digested by trypsin without gel separation and subjected to LC-MS/MS. Per one hundred high scoring overlapping proteins we found only 18 unique cellular proteins incorporated in the viral cores from the MLV Env-pseudotyped virus-infected Sup-T1 (15% of all incorporated host proteins) and 10 proteins (9%) in cores of the virus from Sup-T1 cells infected with VSV-G-pseudotyped NL4-3 strain (Figure 3B; Additional file 1: Table S1). Since the minimal difference in the spectra of host proteins between cores of virions harvested from different cell lines was not less than 20%, and was equal to 42% and 45% for the viral cores from Sup-T1 and activated THP1 cells, respectively, relative to cores from non-activated THP1 cells, we believe that the detected differences between the profiles of cellular proteins in the viral cores from various cell lines are significant.

All the cellular proteins in HIV-1 cores were classified into 31 functional categories (Table 2). The most numerous categories were as follows: RNA-binding proteins (29), components of the cytoskeleton (15) and cytoskeleton regulators (21), chaperones (18), DNA-binding proteins (17), proteins involved in vesicular transport (12) and components of the ubiquitin-proteasome system (9). Although these protein groups were numerous in all preparations, the viruses assembled in different types of producer cells demonstrated diversity in the protein spectra and number of proteins within each group (Figure 3C).

Within the forty two cellular proteins which were present in all core preparations, the spectra of molecular chaperones (7), cytoskeleton components (7), and vesicular transport-associated proteins (5) were the most numerous (Figure 3C4). These functional groups of proteins have been shown to be involved in the folding of viral proteins and HIV-1 virion assembly (reviewed in [12,93]). Some of these proteins (clathrin, transferrin receptor 1, RAB7, RAB5C, EHD4, Hsp70, Hsp90, cyclophilin A, β actin, tubulin α1) are very typical for HIV-1 virions and were registered earlier in the samples of purified viral particles (summarized in the database of Host Proteins in HIV-1 http://web.ncifcrf.gov/research/avp/protein_db.asp). Probably some of these proteins, such as β actin, α and β tubulin, moesin and major vault protein 1 are incorporated into HIV-1 virions non-specifically, due to their close proximity to a budding site, as suggested earlier [4,94], whereas other cellular proteins may be incorporated due to specific interactions with viral proteins, such as Gag and GagPol [11,23,33], viral genomic RNA [8], or tRNA^Lys3^ primer [73]. These host proteins, called “Captives” in a recent review [12], may be involved in the virion assembly and budding process or be important for post-assembly steps of HIV-1 life cycle. Below we attempt to assess the packaging of selected cellular proteins in the cores of HIV-1 virions depending on the type of producer cells.

### Semi-quantitative analysis of selected cellular proteins packaging into the cores of HIV-1 virions assembled in different types of cells

**Figure 4:**
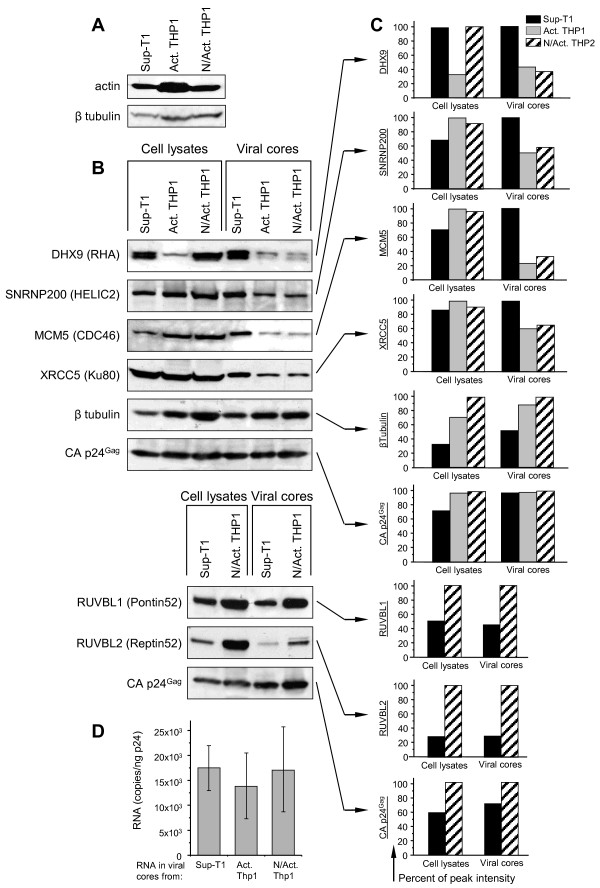
**Incorporation of certain RNA- and DNA-binding cellular proteins into HIV-1 viral cores does not correlate with abundance of these proteins in infected cells. A** – Western blot detection of the cytoskeleton proteins actin and β tubulin in uninfected Sup-T1, activated and non-activated THP1 cells. Lysates were normalized according to cell counts and then according to the count of β globin DNA using quantitative real-time PCR, and subjected to SDS-PAGE and Western blot analysis. **B** – Western blot detection of cellular RNA-binding proteins (DHX9, SNRNP200), DNA- binding proteins (MCM5, XRCC5, RUVBL1, RUVBL2), cytoskeleton protein β tubulin, and viral protein CA p24^Gag^ in the lysates of virus-producing cells (left bands) and in “spin-thru” purified viral cores (right bands). Virus was harvested at 72 h p.i. from Sup-T1, activated and non-activated THP1 cells infected with MLV Env-pseudotyped HIV-1 NL4-3, normalized to CA p24^Gag^ and subjected to the “spin-thru” core isolation. Lysates of infected cells were normalized according to total protein count and β globin DNA count as described in A. Cellular and viral core preparations were analyzed by Western blotting. **C** – Quantification of Western blotting results. Western blotting data were quantified using ImageJ software. Results are presented as percentage of the peak value for each protein in the cellular and viral core preparations. **D** – Quantification of viral genomic RNA in the cores of virions. Viral cores were prepared as described in Figure 1. RNA was isolated from CA p24^Gag^-normalized core samples, subjected to reverse transcription with oligo-dT primer and then to quantitative real-time PCR with the primer set specific for positive-strand HIV-1 DNA. The data represents analysis of three independent preparations. Each point shows mean RNA copy number ± SD per 1 ng of p24^CA^ in the viral core sample.

Although the spectra of the cellular proteins incorporated in the viral cores depended on the type of virus-producing cells, the quantitative differences within the group of overlapping proteins could also be observed. We compared the abundance of certain cellular proteins, selected from the group of 42 common proteins, in the viral cores and lysates of the producer cells. Since the group of 42 cellular proteins identified in all viral cores included the members which are likely involved in viral replication (Table 3), we selected 7 proteins from different functional categories for analysis by Western blot. Within the group of RNA-binding proteins, we analyzed incorporation of RNA helicase A, because the function of this protein in HIV-1 replication has been shown earlier [10,95], and small nuclear ribonucleoprotein 200 kDa (U5) (SNRNP200 or HELIC2), which currently has no known role in HIV replication but has been detected with a high score in our MS/MS preparations. Within DNA-binding group of proteins, we analyzed the Minichromosome maintenance complex component 5 (MCM5) involved in initiation of DNA replication, 80-kilodalton subunit of the Ku heterodimer protein or ATP-dependant DNA helicase II (Ku80 or XRCC5), which is involved in the repair of DNA double-strand breaks and telomerase function [96,97], Pontin52 (RUVBL1) and Reptin52 (RUVBL2) DNA helicases, both being the components of several high molecular weight protein complexes involved in chromatin remodeling, transcription regulation, DNA damage sensing and repair [98]. All these DNA-binding proteins were detected with high scores by LC-MS/MS in the cores of virions from both Sup-T1 and THP1 cells. Beta tubulin was selected as one of the major cytoskeletal proteins found earlier in HIV-1 particles (http://web.ncifcrf.gov/research/avp/protein_db.asp). All preparations were normalized to CA p24^Gag^; the cell lysates were additionally normalized by the cell count and β globin DNA count using quantitative real-time PCR. Cytoskeletal proteins, such as actin or β tubulin, could not be used for normalization of the cell lysates, because they are differently expressed in uninfected Sup-T1 and THP1 cells (Figure 4A).

**Table 3:** **Previously discovered viral proteins with a known role in HIV-1 Replication**^a^

**Protein**^b^	**Gene name**^c^	**Score in virion cores from**^d^			**Known role in HIV-1 replication**^e^
		**Sup-T1**	**Act. THP1**	**N-Act. THP1**	
***DNA Binding: Chromatin Organization, Replication, Topoisomerases***					
Regulator of chromosome condensation 2	RCC2	3.32	1.78	-	RCC proteins interact with Rac1 and Arf6 subnetworks and limit signaling required for membrane protrusion and delivery [99]; RCC2 acts as a Rac1 guanine nucleotide exchange factor (GEF); RCC1 is involved in HIV-1 RNA nuclear export through activation of RanGAP [100] and hence facilitates dissociation of RNA nuclear export complex [101].
***RNA Binding: Structure Organization, Modification, Splicing, Transport***					
Helicase like protein 2 - DEAD/H box polypeptide 3	DDX3X	2.05	-	-	Member of DEAD box RNA helicases that is implicated in alteration of RNA secondary structure such as translation initiation, nuclear and mitochondrial splicing, and ribosome and spliceosome assembly. RNAi knockdown of DDX3 suppresses HIV-1 viral replication [102]; interaction of DDX3 with Rev/CRM1 is important for nuclear export of non-spliced HIV-1 RNA [103].
RNA-dependent helicase p72 - DEAD box polypeptide 17 isoform 1	DDX17	6.36	-	-	Members of ATP-dependent DEAD box RNA helicases, potentially involved in interaction with HIV-1 RNA.
RNA helicase II/Gu protein - DEAD box polypeptide 21	DDX21	-	2.07	-	
ATP-dependent RNA helicase A - DEAD box protein 9	DHX9	6.36	6.09	27.18	RNA helicases that catalyze ATP-dependent unwinding of double-stranded RNA and DNA-RNA complexes; localize in both nucleus and cytoplasm and function as transcriptional regulators; may also be involved in expression and nuclear export of retroviral RNAs, particularly in post-transcriptional regulation of HIV-1 [104]. DHX9 is packaged in HIV-1 particles and contributes to particle assembly and reverse transcription [10]; it also facilitates tRNA^Lys3^ binding and initiation of reverse transcription [73]
Heterogeneous nuclear ribonucleoprotein A1	HNRNPA1	-	1.92	-	RNA binding proteins that complex with heterogeneous nuclear RNA (hnRNA) and are involved in pre-mRNA processing in the nucleus: alternative splicing regulation, polyadenylation, nucleo-cytoplasmic transport and other aspects of mRNA metabolism and transport; hnRNP-A1 is involved in HIV-1 mRNA splicing [105,106]; hnRNP-A2 is found to be important for trafficking of HIV-1 mRNA out of the nucleus and through the cytoplasm [105]; hnRNP H and hnRNP K interact directly with HIV-1 RNA and are involved in alternative splicing [107].
Heterogeneous nuclear ribonucleoprotein F	HNRNPF	3.47	-	-	
Heterogeneous nuclear ribonucleoprotein H1	HNRNPH1	3.04	-	8.26	
Heterogeneous nuclear ribonucleoprotein M isoform b	HNRNPM	1.9	-	--	
Heterogeneous nuclear ribonucleoprotein R	HNRNPR	6.31	-	--	
Nonsense-mediated decay (NMD) factor	UPF1	5.65	6.48	6.32	ATP-dependent RNA helicase, the HIV-1 RNP component, positively influences HIV-1 RNA translatability. Important for stability of unspliced viral RNA and translation of Gag polypeptide in producer cells [108]
***Cytoskeleton***					
β Actin	ACTBL2	60.12	35.05	99.59	Actin microfilaments are important for RTC formation and RTC transport in cytoplasm [1]; interaction with NC domain of Gag is required for HIV-1 assembly [109].
Tubulin α 1	TUBA1A	69.39	58.56	66.49	Microtubules are shown to be important for RTC cytoplasmic trafficking [18,110]assembly of Gag polyprotein molecules [111] and viral genomic RNA trafficking [112].
Tubulin α 6	TUBA1C	57.11	41.39	61.72	
Tubulin β 5	TUBB	29.82	27.85	49.68	
Tubulin β 3	TUBB3	13.84	9.07	30.56	
Tubulin β 1	TUBB1	13.3	-	22.5	
Tubulin γ 1	TUBG1	-	-	4.96	
Dynein	DYNC1H1	-	11.68	10.11	Dynein motor and late endosomes are involved in viral RNA trafficking [112] and transport of RTC toward the nucleus [18].
***Cytoskeleton Regulation***					
ARP3 actin-related protein 3 homolog (yeast)	ACTR3	-	9.52	-	Major constituent of the ARP2/3, a 7 subunit complex, responsible for actin polymerization [113]. The complex is required for early phase of HIV-1 replication [114].
***Nuclear Import***					
Transportin 3; importin-SR; importin 12; transportin SR2	TNPO3	1.0	-	-	Impotin-β family member, binds catalytic core domain close to the N terminus of IN and promotes nuclear entry of PICs [115,116]; might serve as a chaperone that associates with PIC post-entry to guide it through nuclear pore [117].
Karyopherin α2; importin α1)	KPNA2	-	-	7.91	Directly interacts with central core domain of HIV-1 integrase, facilitates PIC nuclear import [118-120].
Karyopherin β2; importin β2	TNPO1	-	3.63	1.95	Importin α/β heterodimer interacts with HIV-1 integrase and probably MA protein and Vpr to translocate PIC into the nucleus [121]
Karyopherin β1; nuclear factor p97; importin 90	KPNB1	-	-	8.02	
***Nuclear Export***					
RANBP21/exportin 5 (Exp5)	XPO5	5.5	-	6.87	Association of RanBP1 and 2 with Rev-CRM1-RanGTP complex has been shown [122], thus RanBP is required for dissociation of nuclear export complex during HIV-1 RNA nuclear export [101].
***Vesicular Transport***					
CLTC protein - clathrin	CLTC	37.6	7.7	7	Clathrin is incorporated in HIV-1 particles probably through interaction with Pol, especially IN domain [32]; it facilitates the accurate morphogenesis of infectious particles probably by contribution to spatial organization of Gag and Pol proteins and proteolytic processing of virion components during particle assembly [5].
Rab5C GTP protease	RAB5C	-	6.78	6.58	Rab GTP proteases are important for vesicular trafficking. They are activated by guanine nucleotide exchange factor (GEF), RCC2 protein revealed in HIV-1 cores can act as a GEF. Rab11 is important for HIV-1 production [123]; Rab1 potentially associates with HIV-1 Rev and is involved in nuclear export of viral RNA [124]; Rab9 is required for Gag trafficking to the site of assembly [123]; Rab7-interacting lysosomal protein promotes vRNA clustering at the MTOC [112]; Rab6 is probably involved in viral entry [58].
Rab7A GTP protease	RAB7A	8.89	9.08	21.97	
Rab8A GTP protease	RAB8A	7.91	6.78	6.58	
Rab11A GTP protease	RAB11A	-	-	9.31	
Programmed cell death 6 interacting protein; HP95; AIP1/ALIX	PDCD6IP	6.43	13.62	29.94	Alix/HP95 is a protein implicated in endosomal organization and virus budding; overexpression results in cytoplasmic vacuolization, which may be partially responsible for protection against cell death. AIP1/ALIX is a binding partner for HIV-1 Gag L-domain and other budding network proteins (Tsg101) functioning in virus budding [125].
***Intracellular Trafficking***					
TNFAIP3 interacting protein 1; HIV-1 Nef interacting protein; NAF1	TNIP1	1.0	-	-	An ERK2 binding protein, Naf1, attenuates EGF/ERK2 nuclear signaling, binds HIV-1 Nef and increases cell surface CD4 expression [126]. ERK2 interacts with HIV-1 matrix, packaged into virions and responsible for MA phosphorylation [24].
***Chaperones/Molecular Folding***					
Hsp70 protein 8	HSPA8	7.85	26.4	31.35	Heat shock protein 70 family members are shown to be incorporated in HIV-1 particles. This is important for subsequent viral cDNA synthesis [11,127]; they can also interfere with Vpr in HIV-1 nuclear import in macrophages [128,129].
Hsp70 protein 9 (mortalin)	HSPA9	-	5.75	-	
Cyclophilin A, CyPA	PPIA	22.35	20.00	7.68	incorporates into virions via binding to the CA domain of Pr55Gag [23]. The role of CA-bound CyPA is still unclear [48] It is critical for protection and stabilization of HIV-1 cores as a chaperone [49] and is probably involved in PIC nuclear transport [31]

^a^ Proteins are listed by function category.

^b^ Full Protein name as listed in NCBI Protein database (http://www.ncbi.nlm.nih.gov/protein).

^c^ Official Gene Symbol as listed by HGNC.

^d^ The Xcorr values of each protein in different viral core samples are shown; if protein was not present in the virion core from a specific producer cell type, the Xcorr is not shown.

^e^ Protein function within the cell is listed, along with known implicated function(s) in HIV-1 replication.

Analysis of selected proteins using Western blot with subsequent quantification of the band intensity using ImageJ software did not reveal differences in the abundance of β tubulin between the infected cells and the cores of virions produced by these cells (Figure 4B,C). The same ratio of protein concentrations between cells and viral cores was also detected for RUVBL1 and RUVBL2 DNA helicases. However, other analyzed DNA- and RNA-binding proteins demonstrated either moderate (XRCC5, SNRNP200) or high prevalence (MCM5, DHX9) in the cores of virions assembled in Sup-T1 cells, whereas in the cores of THP1-derived virions (both activated and non-activated) these proteins were less abundant. Incorporation of these proteins in the viral cores did not correlate with their abundance in the producer cells: XRCC5, MCM5 and SNRNP200 were presented in all types of cells at a similar level, whereas the RNA helicase A (DHX9) was abundant in dividing Sup-T1 and non-activated THP1 cells and decreased in non-dividing activated THP1 (Figure 4B,C). Interestingly, the incorporation of RNA helicases DHX9 and SNRNP200 into the cores did not correlate with the packaging of viral genomic RNA, as quantitative RT-PCR of the RNA isolated from CA p24^Gag^-normalized purified viral cores revealed no significant differences in the count of HIV-1 RNA in the cores from Sup-T1 and both THP1-derived viruses (Figure 4D).

## Discussion

The proteomic analysis of the cores isolated from HIV-1 virions assembled in T lymphocytes and activated (model of MDM) and non-activated monocytic cells revealed more than thirty cellular proteins which have been previously shown to be involved in different steps of HIV-1 replication and/or incorporated in HIV-1 virions (Table 3). Since HIV-1 is a retrovirus with a RNA genome of less than 10 kB and encodes only nine polypeptides, it engages numerous cellular factors and pathways at all stages of its life cycle. Some of the factors, especially proteins involved in RNA splicing and nuclear export, multi-vesicular bodies (MVB) and late endosomal pathway, as well as the proteins directly involved in the HIV-1 budding process, such as AIP1/Alix, may be incorporated into virions by being associated with the viral RNA and proteins, but do not play a visible role in subsequent stages of viral replication [4,125,130]. For the other factors, such as Hsp70, CLTC protein/clathrin and Rab GTP proteases, their important role in the molecular organization of mature virions and probably viral entry into the target cells has been proposed earlier [5,11,32,33,58]. Here, we focus on the proteins identified in the core structures of HIV-1 virions assembled in different cell types. These proteins can be potentially involved in post-entry stages of the viral replication.

The HIV-1 morphogenesis is known to be different in T lymphocytes and myeloid cells. In T cells, the viral particle budding and assembly have been shown to take place directly at the plasma membrane (reviewed in [131]), whereas in macrophages, earlier studies detected assembling HIV-1 particles in the late endosomes [132] (reviewed in [131]) or in internally sequestered plasma membrane domains that contain late endosomal markers but are connected to the cell surface [133]. Recent reports revealed an extensive tubular network and large sheet-like structures which extended to the cell surface from vesicular compartments and contained HIV-1 virions, released into the extracellular media [134,135]. Indeed, the cores of model MDM-derived virions contained twelve vesicular trafficking-associated proteins, whereas only six were detected in the T cell-derived viral cores. The larger proportion of cytoskeleton and cytoskeleton regulatory proteins in the cores of virions assembled in THP1 cells than in Sup-T1-derived cores may be dependent on the abundance of these proteins in producer cells. Uninfected THP1 cells contain larger amounts of actin and β tubulin than Sup-T1 (Figure 3A), suggesting that other cytoskeleton and associated cytoskeleton regulatory proteins may also be more abundant in these myeloid cells; hence, increasing the probability that the virus will hijack this subset of proteins. Our data suggest that the unique core-incorporated proteins, which are different in the viruses assembled in different cell types, are mostly indiscriminately hijacked during virion assembly and likely not important for subsequent infection.

The group of forty two cellular proteins identified in the cores of virions produced by all types of cells contains at least thirteen proteins whose involvement in different stages of HIV-1 infection has been shown previously (Table 2 and 3). On the other hand, many proteins within this group have never been found to be implicated in any infection event. Meanwhile, incorporation of these proteins into the viral cores from different types of producer cells suggests that at least some of them may be important for successful infection. For instance, within the functional category of vesicular trafficking-associated proteins, the member of ESCRT pathway AIP1/ALIX detected in all our core preparations has been shown earlier to be interacting with HIV-1 Gag late domain and to be important for the release of viral particles [125,136]. Clathrin has also been found to be abundant in HIV-1 viral particles and important for the correct assembly and maturation of viral particles through the regulation of proteolytic processing of virion components [5,32]. Members of the RAB family of proteins were found to be important for different steps of HIV-1 particle assembly and probably RNA incorporation [112,123,124] (Table 3). Available data suggest that the proteins of this group are important factors of assembly and maturation of the viral particle and get into the viral cores in association with Gag and GagPol proteins or viral RNA. Some of the factors of vesicular trafficking may be potentially involved in the early stage of HIV-1 infection: the functional genomic screening of factors involved in HIV-1 infection showed that the vesicular RAB6A protein is important for the late phase of reverse transcription in infected cells [58].

The cytoskeletal proteins were also abundant in all core preparations, however, the role of these proteins in HIV-1 virions is still questionable. The actin microfilaments form the plasma membrane cortex, and both actin and microtubular networks are involved in HIV-1 particle assembly [111,112,137], so that the proportion of these proteins in viral particles may be up to 15% of the molar level of Gag [94]. Actin is packaged in the virions probably in association with the NC domain of Gag [111,137,138]. Thus, actin and actin-associated proteins coronin, moesin, filamin, and FLII can get into the viral cores due to the actin interaction with Gag or GagPol. However, involvement of these virion-packaged molecules in post-assembly events of the virus life cycle has not been shown [12]. Our data indicate that the ratio of β tubulin between the viral cores reproduced the concentration ratio of this protein between the producer cells, suggesting the capturing, but not specific incorporation, of this cytoskeletal protein into assembling virions. On the contrary, the cytoskeleton regulation proteins were found to be mostly different in the viral cores from different cell types, which may reflect variability of the profiles of these proteins in virus-producing cells. Some of them, particularly Hsp27, a protein containing a nuclear localization signal [139] and found in the cores of virions from all cell types, can be potentially involved in post-entry steps of infection, although the role of this protein in viral replication remains unknown.

The other category of proteins abundant in HIV-1 viral cores is the molecular chaperones. The profiles of these proteins are very similar in all analyzed samples. Indeed, Hsp27, Hsp40 (DnaJ) co-chaperone, Hsp70, Hsp90, numerous members of TCP1 (Hsp60) tetradecameric complex, as well as peptidylprolyl isomerase cyclophilin A were found in all core preparations (Table 2). Previously, these proteins were identified in purified samples of whole HIV-1 virions and Gag preparations [4,10,23]. Hsp70 was also found in HIV-2, SIV_MAC_ and SIV_AGM_[11,56]. Since the major function of these proteins is to regulate folding of newly synthesized polypeptides, facilitate intracellular protein transport and assemble multisubunit protein structures [140,141], they likely play an important role in HIV-1 particle assembly, processing and folding of the viral proteins during virion core maturation and maintain structural integrity of the viral core and RTCs [11,33,49]. The early RTC functions, especially organization of reverse transcription, may also depend on the proper activity of incorporated chaperones.

The RNA-binding proteins represent the most diverse group of cellular factors in viral cores. Although we identified 29 RNA-binding proteins in the core preparations, only four of them were found in all core samples (Table 2). These are (1) regulator of nonsense transcript stability (UPF1), (2) ATP-dependent RNA helicase A (RHA or DHX9), (3) small nuclear ribonucleoprotein component (Snrp116 or EFTUD2), and RNA helicase hBrr2 200 kDa (SNRNP200 or HELIC2). The role of the first two factors in HIV-1 infection was thoroughly characterized before. The UPF1 protein, RNA helicase from the SFI superfamily, involved in translation of Gag polypeptide, was found in virus-producing cells in association with HIV-1 ribonucleoprotein (RNP) along with Pr55Gag, viral RNA and cellular protein Staufen 1 [108]. Thus, UPF1 can be packaged in the virions in association with both Gag and the viral genomic RNA. RNA helicase A (RHA), a member of the DEAD family of proteins which are capable of unwinding the double-stranded RNA structure, was earlier found to be associated with HIV-1 Gag and incorporated into HIV-1 virions in an RNA-dependent manner. Packaging of this protein into HIV-1 virions was important for endogenous reverse transcription [10]. Jeang and Yedavalli suggested that RHA incorporated into HIV-1 viral cores might be important for the reverse transcription in RTCs [95]. A recent study that revealed an important role of this enzyme in the annealing of tRNA^Lys3^ primer [73], confirmed this suggestion. Two other proteins, SNRNP200 (member of the family of U5 DEXH-box RNA helicases) and Snrp116 (U5 snRNP specific protein, 116 kD), are both members of the U5 group of small nuclear RNA proteins, the spliceosome components, and have not been detected in HIV-1 virions before. Since these proteins are known as important components of splicing machinery required for a spliceosome catalytic activity [142,143], they can be associated with HIV-1 pre-mRNA and remain associated with a mature viral RNA molecule.

Interestingly, our analysis showed higher level of DHX9 (RHA) and SNRNP200 (HELIC2) in the cores of virions assembled in T cells, as compared with the viral cores from the monocyte and MDM models, which did not correlate with the abundance of these proteins in producer cells. Since we did not find significant differences in the RNA and CA protein count between the virions from analyzed cells, observed differences suggest that the mechanism of incorporation of these proteins into the virions (binding to viral RNA or interaction with Gag or/and GagPol) is more effective and likely selective in T lymphocytes, than in monocyte and MDM model cells. Because of importance of RNA helicase A for the reverse transcription in HIV-1 virions and RTCs, we expect that SNRNP200 protein may also be involved in cDNA synthesis or accumulation.

Analysis of earlier published genome-wide screens performed by Warrilow and co-authors [22] to select the host factors potentially implicated in HIV reverse transcription showed that the proteins involved in DNA replication, transcription and repair, as well as proteins of the ubiquitin-proteasome pathway may also be important. Within the 17 DNA-binding proteins detected in our core preparations, only two, ATP-dependent DNA helicase II (XRCC5 or Ku80) and TATA binding protein interacting protein 49 kDa (RUVBL1 or Pontin52), were found in the core of all virions. Two other proteins, minichromosome maintenance complex component 5 (MCM5 or CDC46) and regulator of chromosome condensation protein 2 (RCC2), were identified only in Sup-T1 and activated THP1 cells (Table 2), although Western blot showed presence of MCM5 also in non-activated THP1. The protein RUVBL2 (Reptin52) was not identified by MS/MS in activated THP1, but the fact that in cells this DNA helicase is complexed with the closely related RUVBL1 protein in hetero-dodecamers [144] suggests incorporation of this protein in the virions from all studied cells. Our analysis showed that among DNA binding proteins present in the viral cores from different cell types only XRCC5 and especially MCM5 displayed an increased level of incorporation from the T cells, similar to RNA-binding DHX9 and SNRNP200, whereas core incorporation of RUVBL1 and RUVBL2 reproduced their level in virion-producing Sup-T1 and THP1 cells. The DNA helicase MCM5, a member of the MCM family of chromatin-binding proteins is involved in the initiation of DNA replication and was found to be upregulated during the transition from the G0 to G1/S phase of the cell cycle (RefSeq database). Interaction of this protein with HIV has not been shown before. Another DNA helicase, XRCC5 or Ku80, which is involved in repairing DNA double-strand breaks, was earlier found to be important for viral cDNA circularization, nuclear import and integration [145,146]. However, this function was shown for the protein expressed in the infected target cells, but not for virion-incorporated Ku80. Packaging of both MCM5 and XRCC5 (Ku80) DNA helicases in all viral cores and their high levels in the cores of T lymphocyte-derived virions suggest that the core-incorporated molecules of these proteins can also be involved in processes associated with cDNA processing and/or integration during post-entry steps of infection, especially in T cells.

Within the group of ubiquitin-proteasome pathway associated proteins a total of 9 proteins were detected; three of them were identified in viral cores from all producer cells (Table 2). Earlier, numerous 26 S proteasome-associated proteins were found in HIV-1 and SIV particles [4,56]. Involvement of the ubiquitin-proteasome system in the budding of lentiviral particles was shown earlier (reviewed in [147]). Since all major domains of the membrane-associated HIV-1 Gag molecules have been shown to be ubiquitinated during virion budding [148], the ubiquitination factors could package into virions in association with Gag and then get into the viral cores. However, the role of virion-associated factors of the ubiquitin-proteasome system in the early steps of HIV-1 infection is unknown.

## Conclusions

Taken together, results of our study indicate that the profile of host cell proteins packaged in the cores of HIV-1 virions depends on the type of producer cell. High abundance of certain proteins in the cell increases the probability of their capturing by the virions and hence their presence in the viral cores. However, certain members of functional groups of DNA- and RNA-binding proteins, molecular chaperones, cytoskeletal, vesicular trafficking-associated and ubiquitin-proteasome pathway-associated proteins were found in the cores of virions from all analyzed cells, suggesting that their incorporation is non-random and that they can be directly or indirectly involved in either the virus assembly/budding or early infection events. Our findings that the abundance of cellular proteins DHX9, (RHA) SNRNP200, MCM5, and XRCC5 (Ku80) within virus-producing cells did not correlate with the abundance seen in cores of produced virions, specifically their unexpected higher packaging in T cells, suggests that the incorporation of these factors in T lymphocytes is more efficient than in myeloid cells. These differences may be associated with variability of localization of these host proteins relative to the sites of virion assembly in different cell types and/or with different localization of virion assembly complexes. The host factors abundant in the viral cores may play a role in subsequent steps of HIV-1 infection, specifically in T cells. Further analysis of the role of these proteins in viral replication might reveal new mechanisms of the modulation of HIV infection by the host proteins and identify new targets for antiretroviral therapeutic interventions.

## Methods

### Cells and viruses

The acute monocytic leukemia cell line THP1 (from S. Tsuchiya) and T lymphoblastoma Sup-T1 cells (from James Hoxie) were provided by the NIH AIDS Research & Reference Reagent Program. The human kidney fibroblasts 293 T/17 was purchased from ATCC (Manassas, VA). All cells were maintained at 37°C and 5% CO_2_ in 75 cm^2^ tissue culture flasks with RPMI-1640 culture media supplemented with 10% Fetal Bovine Serum, penicillin/streptomycin (100 μg/ml), and L-Glutamine.

The stocks of the HIV-1 virus pseudotyped with amphotropic murine leukemia virus envelope glycoprotein (MLV Env) for synchronized infection of THP1 and Sup-T1 cells were prepared by transfection of 293 T/17 cells with HIV-1 NL4-3 provirus-encoding plasmid [149] and pcDNA-Env(MLV) plasmid (kindly provided by Nathaniel Landau) at a 4:1 ratio using Metafectene transfection reagent (Biontex, Planegg, Germany). The cells were transfected in 75 cm^2^ tissue culture flasks; the plasmid DNA containing media was changed at 5 h post-transfection. After overnight incubation with fresh RPMI-1640, the media was changed again and the cells were cultured in fresh media for an additional 48 h at 37°C and 5% CO_2_. Then, the supernatants were harvested, filtered through a 0.45-μm filter and stored on wet ice at 4°C for 1–4 days.

### Infection

The viral suspensions were normalized according to their RT activity corresponding to 1 × 10^6^ cpm per 1 × 10^6^ cells, mixed with Polybrene (Sigma) to a final concentration of 8 μg/ml and used for infection of approximately 200 × 10^6^ viable Sup-T1 or THP1 cells by spinoculation [150]. Infection was performed in 6-well plates (5 × 10^6^ cells per well) by centrifugation at 1000xg for 2 h at 18°C. After a 2 h incubation at 37°C and 5% CO_2_, the cells were washed from the virus-containing media, re-suspended in RPMI-1640 (pre-warmed to 37°C) and seeded in a regular (Sup-T1 and non-activated THP1) or polylysine-treated (THP1 for activation) 75 cm^2^ tissue culture flasks at a concentration of 4 × 10^6^ cells per ml (Sup-T1, non-activated THP1) or of 1 × 10^6^ cells per ml (THP1 for activation). To get activated THP1 cells, the PMA and vitamin D_3_ solutions were added to cells to a final concentration of 100 nM. Then, the cells were incubated at 37°C and 5% CO_2_ for 72 h.

### Concentration of virus and “spin-thru” isolation of viral cores

Virus-containing culture media from infected Sup-T1, activated and non-activated THP1 cells, as well as the media from the same types of non-infected cells (control) were harvested at 72 h after incubation with virus (or equivalent volume of the virus-negative culture media) and purified from cell debris by being centrifuged at 2,500 rpm and 4°C for 5 minutes and filtered through 0.45 μm syringe filters. Then, filtered samples were centrifuged at 100,000xg and 4°C for 3 h through 2 ml cushions of 30% sucrose in STE buffer (10 mM Tris–HCl [pH 7.4], 100 mM NaCl, and 1 mM EDTA) in a Beckman SW-41 rotor. The pellets were re-suspended in 300 μl of STE buffer and the viral cores were then isolated by “spin-thru” purification as described earlier [63-66]. Briefly, 3.8 ml of a 30-50% linear density gradient of sucrose in STE buffer was overlaid with 1 ml of 15% sucrose containing 1% Triton X-100 and then covered with a 0.4-ml cushion of 7.5% sucrose in STE. The HIV-1 positive and negative samples, concentrated through 30% sucrose and resuspended in STE (0.3 ml) were carefully layered on top of the 7.5% sucrose layer and centrifuged in a Type 100 Ti rotor (Beckman Coulter) at 100,000xg and 4°C for 16–18 h. The pellets were re-suspended in 26 μl of STE buffer and replaced to polypropylene, non-siliconized Eppendorf microtubes; 4 μl aliquots were set aside for the p24^CA^ ELISA assay. The CA p24^Gag^–normalized suspensions of HIV-1 cores and control suspensions were subjected to SDS-PAGE protein separation for subsequent LC-MS/MS analysis, Western blotting, or to In-solution protein digestion with trypsin for the LC-MS/MS analysis of unseparated protein samples.

In order to test purity of the “spin-thru” isolated cores from undestroyed viral particles, 400 μl aliquots of the suspensions of viral cores and concentrated whole virions were separately subjected to centrifugation in a 30-70% sucrose gradient for 5 h at 125,000xg and 4°C in a SW-60Ti rotor (Beckman Coulter). Ten fractions of the gradient (each 400 μl) were then collected from the bottom of the tubes and densities were determined. All fractions were dialyzed versus 1 L of ice-cold PBS using Tube-O-DIALYZER 1 kDa MEDI Kit (G Biosciences, St. Louis, MO) according to the manufacturer’s protocol and then applied for p24 enzyme-linked immunosorbent assay using Alliance HIV-1 p24 ELISA Kit (PerkinElmer, Waltham, MA).

Additionally, electron microscopy (EM) was applied to test purity of the viral and core preparations. For EM, the virions concentrated through 30% sucrose and “spin-thru”-purified core preparations were resuspended in 20 μl of STE buffer, incubated 20 minutes on formware carbon film-coated 100 square mesh nickel grids (Electron Microscopic Sciences) at room temperature, and then incubated with 4% glutaraldehyde fixing solution for 10 minutes. After five-time wash in molecular grade water (Mediatech, Manassas, VA), samples were stained with 2% uranyl acetate. For analysis of virion structure, the pellets of virions after centrifugation through 30% sucrose were washed twice with PBS and then fixed with 4% glutaraldehyde for 4 h. The preparations were further fixed with 2% OsO_4_ for 2 h, dehydrated with a graded series of ethanol dilutions ranging from 25% to 100% and then embedded in Araldite 502 resin. Ultrathin sections were contrasted with 2% uranyl acetate in methanol and 1% lead citrate. All preparations were examined on a JEOL JEM 1200 transmission electron microscope operating at 100 kV.

### Western blot analysis

The aliquots of the lysates of HIV-1 infected Sup-T1 and THP1 cells, the virus samples and culture media from non-infected cells taken before and after the “spin-thru” isolation were subjected to SDS-PAGE, subsequently transferred to a PVDF membrane and then detected using anti-HIV-1 p24 (24–3) mouse monoclonal antibody and human HIV immunoglobulin (HIV-IgG) from NIH AIDS Research & Reference Reagent Program; anti-RNA Helicase A (ab70777) rabbit polyclonal antibody from Abcam; anti-CD45, clone F10-89-4 monoclonal antibody from Millipore (Temecula, CA); monoclonal anti-Actin clone AC-40 from Sigma; anti-β tubulin (D-10), anti-Reptin 52 (D-6), anti-Ku80 (B-1) and anti-MCM5 (G-1) mouse monoclonal antibodies from SantaCruz Biotechnology (Santa Cruz, CA); anti-HELIC2 (N-20) and anti-Pontin 52 (N-15) goat polyclonal antibodies also from Santa Criz. Specific bands were visualized by ECL (Thermo Scientific, Rockford, IL). Quantification of the Western blotting results was performed using ImageJ software.

### Gel separation of proteins, protein digestion and peptide extraction

The volumes of viral core suspensions, each containing 400 ng of p24^CA^ protein, and control suspensions taken in twofold excess were mixed with equal volumes of Laemmli Sample Buffer (BioRad, Hercules, CA) containing 5% β mercaptoethanol, heated in boiling water for 2 minutes and applied for SDS-PAGE protein separation. Separation of proteins was performed in 12.5% Tris–HCl Criterion Precast Gel (BioRad) at 100 V and 4°C for 2–2.5 h. The gel was stained in 0.1% (wt/v) Coomassie (BioRad) solution (40% methanol (v/v), 10% acetic acid (v/v) in water with 1 g/L of Brilliant Blue R-250) for 1 h at room temperature. After 7–8 washes in de-staining solution (contains the same components, as staining solution, except Brilliant Blue R-250) the gel was replaced to water, and each lane was sectioned into 10 contiguous pieces, which were subjected to the “in-gel” proteolysis according to the modified previously published protocol [151] Briefly, acetonitrile (ACN) dehydrated gel pieces were rehydrated in 10 mM DTT and incubated at 60°C for 1 h. After cooling at room temperature, the gel slices were incubated with 50 mM iodacetamide for 1 h at room temperature in the dark for alkylation of proteins. After the second dehydration, a 15 μl dose of Trypsin Gold (Promega, Madison, WI) solution (20 μg/ml) in 40 mM NH_4_HCO_3_/10% ACN was added to each of the gel pieces. After 1 h saturation at 4°C, the pieces were incubated at 37°C overnight. The resulted peptides were extracted three times: (1) with 25 mM of NH_4_HCO_3_:ACN (1:1); (2) 5% formic acid (FA); (3) 5% FA:ACN (1:1). After pooling all the extracts together, samples were purified through ZipTip pipette tips C18 (Millipore), eluted with 30 μl of 0.1% trifluoroacetic acid (TFA) in 80% ACN and subjected to HPLC separation and MS/MS analysis.

For “in-solution” protein digestion, the suspensions of HIV-1 cores after “spin-thru” centrifugation were treated with 10 mM DTT (60°C for 1 h) and 150 mM iodacetamide (1 h at room temperature in the dark) in 20 ul of STE buffer. The protein samples were then mixed with 100 μl of 200 mM ammonium bicarbonate and treated with 200 ng of Trypsin Gold (Promega) at 37°C overnight. The resulted peptides were dried in SpeedVac, resuspended in water, purified through ZipTip pipette tips C18 as described above and then subjected to HPLC separation and MS/MS analysis.

### HPLC-MS/MS of tryptic digests and database search

The peptides in each sample were separated by micro-capillary reversed-phase liquid chromatography (HPLC), coupled online to an ion trap mass spectrometer Thermo LTQ Orbitrap XL. The mass spectrometer was operated in a data-dependent MS/MS mode using a normalized collision-induced dissociation (CID) energy of 35%. The CID spectra were compared against those of the EMBL non-redundant protein database. Only peptides having cross-correlation (*X*_corr_) cutoffs of 2.6 for [M + 2 H]^2+^, 3.0 for [M + 3 H]^3+^ and higher charge state were considered. These SEQUEST criteria thresholds resulted in a 1-2% of False Descovery Rate. The proteome analysis of the spectra was made by Proteome Discoverer 1.2 software (Thermo Fisher Scientific). The protein profiles of the samples of viral cores were compared with identically prepared samples from non-infected cells. The sub-cellular localization and function of each filtered protein was determined using gene ontology (GO) information obtained from cross-referencing each protein’s Swiss-Prot accession number to the GO localization information available on the NCBI protein database (http://www.ncbi.nlm.nih.gov/sites/entrez?db=Protein) and The Human Protein Atlas database (http://www.proteinatlas.org). The involvement of the proteins in known cellular pathways associated with major biological processes such as cell cycle, intracytoplasmic transport, cytoplasm organization, nuclear transport, chromatin structure maintenance/regulation, RNA splicing and reorganization, transcription, apoptosis, proteasomal degradation, etc. were assessed using NCBI RefSeq database (http://www.ncbi.nlm.nih.gov/RefSeq/) and DAVID Bioinformatics Resources 6.7 (NIAID NIH) (http://david.abcc.ncifcrf.gov).

### RNA purification and RT reaction

RNA was purified from suspensions of “spin-thru” purified viral cores containing 250 ng of p24^CA^ using TRI Reagent-LS (MRC, Cincinnati, OH) according to the manufacturer's protocol. A total of 0.5 μg of RNA from the RNA fraction was treated with 0.25 mg/ml DNase I RNase-free (Roche, Mannheim, Germany) for 60 minutes in the presence of 5 mM MgCl_2_, followed by the heat inactivation at 65°C for 15 minutes. A 250 ng aliquot of total RNA was used to generate cDNA with the GoScript Reverse Transcription System (Promega, Madison, WI) using oligo-dT reverse primers.

### DNA isolation and quantitative real-time PCR

Lysates of HIV-1 infected (72 h p.i.) Sup-T1, activated and non-activated THP1 cells were normalized to the total protein count using DC Protein Assay (BioRad) following manufacturer’s protocol. The total DNA was isolated using an IsoQuick Nucleic Acid Extraction Kit (ISC BioExpress, Kaysville, UT) following manufacturer’s recommendations. After isolation, the cellular DNA samples were analyzed by quantitative TaqMan real-time PCR to quantify chrosomal DNA. Set of primers specific for the β-globin gene has been used: forward primer BGF1 (5’-CAACCTCAAACAGACACCATGG-3’), reverse primer BGR1 (5’-TCCACGTTCACCTTGCCC-3’), and probe BGX1 (5’-FAM-CTCCTGAGGAGAAGTCTGCCGTTACTGCC-TAMRA-3’). The 2 μl aliquots of RT reaction mixtures of the RNA samples from isolated viral cores (see above) were diluted to 10-fold and 100-fold and subjected to quantitative real-time PCR analysis with the set of primers specific for late HIV-1 reverse transcription product as described earlier [152]. The primers FOR-LATE (5’-TGTGTGCCCGTCTGTTGTGT-3’), REV-LATE (5’-GAGTCCTGCGTCGAGAGATC-3’), and probe Lt-LTR-Prb (5’-FAM-CAGTGGCGCCCGAACAGGGA-TAMRA-3’) recognized the positive-strand DNA, specific for the U5-Ψ LTR region. PCR reactions were performed with PerfeCTa qPCR FastMix, UNG (Quanta Biosciences, Gaithersburg, MD) using 300 nM of each primer and 200 nM of probe according to the manufacturer protocol. Serial dilutions of DNA from 8E5 cells (CEM cell line containing a single copy of HIV-1 LAV provirus per cell) were used as the quantitative standards. Real-time PCR reactions were carried out at least in triplicate using the PTC-200 Peltier Thermal Cycler with Chromo4 Continuous Fluorescence Detector (both from MJ Research) and Opticon Monitor 2.03 software.

## Competing interests

The authors declare that they have no competing interests.

## Author’s contributions

SS participated in the design of experiments, carried out most of the experiments, prepared samples for mass spectrometry and electron microscopy, analyzed data and contributed to manuscript preparation. YO performed LC-MS/MS data collection and analyzed raw mass spectrometry data. SN participated in the design of the study, supervised LC-MS/MS experiments and contributed to drafting of the manuscript. MB participated in the study design and coordination and contributed to manuscript preparation. SI conceived of the study, designed and coordinated experiments, participated in data analysis and prepared the manuscript. All authors read and approved the final manuscript.

## Supplementary Material

Additional file 1 **Table S1:** Overlapping and unique high scored cellular proteins within viral cores isolated from the virus produced by Sup-T1 cells infected with HIV-1 NL4-3 strain pseudotyped with MLV Env (blue symbols) or VSV-G (green symbols) envelope glycoproteins. Click here for file
